# Training and support for the role of facilitator in implementation of innovations in health and community care: a scoping review protocol

**DOI:** 10.1186/s13643-023-02172-x

**Published:** 2023-01-31

**Authors:** Malin Tistad, Anna Bergström, Marie Elf, Leif Eriksson, Catharina Gustavsson, Camilla Göras, Gill Harvey, Ann-Sofie Källberg, Ann Rudman, Maria Unbeck, Lars Wallin

**Affiliations:** 1 School of Health and Welfare, Dalarna University, Falun, SE 791 88 Sweden; 2 Department of Neurobiology, Care Sciences and Society, Karolinska Institutet, Huddinge, Sweden; 3 Uppsala Global Health Research on Implementation and Sustainability (UGHRIS), Department of Women’s and Children’s Health, Uppsala University, Akademiska sjukhuset, Uppsala, SE 751 85 Sweden; 4 Center for Clinical Research Dalarna–Uppsala University, Nissers väg 3, Falun, SE 791 82 Sweden; 5 Department of Public Health and Caring Science, Uppsala University, Uppsala, Sweden; 6 Caring Futures Institute, College of Nursing and Health Sciences, Flinders University, Adelaide, Australia

**Keywords:** Facilitation, Facilitator, Implementation science, Training, Support, Supervision, Healthcare services, Community care, Evidence-based practice, i-PARIHS

## Abstract

**Background:**

Implementing and sustaining innovations in clinical practice, such as evidence-based practices, programmes, and policies, is frequently described as challenging. Facilitation as a strategy for supporting implementation requires a facilitator, i.e. an individual with a designated role to support the implementation process. A growing number of studies report that facilitation can help tackle the challenges in implementation efforts. To optimise the potential contribution of facilitation as a strategy to improve the implementation of new practices, there is a need to enhance understanding about what training and support is required for individuals in the facilitator role.

The objective of this scoping review is to map how facilitators have been trained for, and supported in, the facilitator role in implementation studies in health and community care. Specifically, the review aims to examine what is reported on training and support of facilitators in terms of learning outcomes, content, dose, mode of delivery, learning activities, and qualifications of the trainers and how the facilitators perceive training and support.

**Methods:**

This scoping review will follow the guidance of the Joanna Briggs Institute and the PRISMA Extension for Scoping Review checklist. We will include articles in which (a) facilitation is deployed as an implementation strategy, with identified facilitator roles targeting staff and managers, to support the implementation of specified innovations in health or community care, and (b) training and/or support of facilitators is reported. We will exclude articles where facilitation is directed to education or training in specific clinical procedures or if facilitation supports the implementation of general quality improvement systems. All types of peer-reviewed studies and study protocols published in English will be included. A systematic search will be performed in MEDLINE (Ovid), Embase (embase.com), Web of Science Core Collection, and CINAHL (Ebsco).

**Discussion:**

The proposed scoping review will provide a systematic mapping of the literature on the training and support of implementation facilitators and contribute useful knowledge within the field of implementation science to inform future facilitation initiatives.

**Systematic review registration:**

Registered at Open Science Framework (registration https://doi.org/10.17605/OSF.IO/M6NPQ).

## Background

Implementing and sustaining innovations in healthcare practice, such as evidence-based methods, programmes, and policies, is frequently described as challenging. To overcome this challenge, facilitation is increasingly employed as a strategy to support the implementation of innovations in health and community care. There is a growing evidence base that suggests facilitation can be helpful in successfully tackling the challenges that most often accompany implementation efforts [1–7]. In line with Rogers, we define *innovations* as practices perceived as new by individuals or groups of people [8].

In a compilation of implementation strategies, *facilitation* is defined as “a process of interactive problem solving and support that occurs in a context of a recognised need for improvement and a supportive interpersonal relationship” [9]. The authors of the integrated-Promoting Action on Research Implementation in Health Services (i-PARIHS) framework describe facilitation as the active ingredient of implementation, implying a dynamic and deliberate process to support implementation [10]. As such, facilitation involves assessing and responding to the characteristics of the innovation, the intended recipients of the innovation, and the context in which implementation will be undertaken. Consequently, facilitation requires a facilitator, i.e. one or more individuals with a designated role and the use of enabling facilitation strategies to support the implementation process.

Facilitators can be internal or external to the setting where implementation occurs and a combination of the two is frequently reported [11]. Facilitators are expected to have specific characteristics and skills [12], whilst facilitation is described as a technique that can be learned and improved. The i-PARIHS framework describes three levels of facilitators—novice, experienced, and expert—recognising the complexity of the role and the experiential learning that occurs over time [10]. A recent study underlines the challenge of acting as a facilitator by reporting on the wide range of complex skills required for successfully enacting the role [13]. The advantage of possessing the required facilitation skills, characteristics, and attributes is underlined by the findings of a municipality-based cluster randomised trial on neonatal health and mortality in Vietnam. Municipalities with facilitators assessed as having “higher” scores on appropriate characteristics and skills were significantly associated with a lower neonatal mortality rate [1, 2]. A lack of appropriate skills, and a mismatch between skills and the context for facilitation activities, can compromise how facilitation is carried out, leading to minimal or no impact on outcomes [14, 15]. This suggests that individuals’ training, preparation, and support to take on an implementation facilitator role is an important consideration.

However, detailed reporting of facilitator training and support strategies in previous systematic reviews on facilitation is relatively limited. In Baskerville et al.’s systematic review of facilitation in primary care settings (2012), information is reported on facilitators’ professional and academic background, along with some details on the length of training to be a facilitator (varying between 14 h and 3 months), but nothing further relating to the content of the training. In a scoping review by Cranley et al. on facilitation roles [11], the authors concluded that external facilitators tended to be formally trained for their role, whilst training of internal facilitators varied. Of the 24 intervention studies in the review that reported facilitator training, only 13 provided details on the content of the training. The duration of reported training ranged from 4 h to 7.5 months, but details regarding the training content were simply summarised as covering both theoretical knowledge and skills training. To optimise the potential contribution of facilitation to improving the implementation of new practices, there is a need to better understand the specific training and support required for individuals to function effectively in the facilitator role. This is a gap in the existing literature, which we propose to address through the scoping review outlined here.

The Canadian Institutes of Health Research state that scoping reviews are “exploratory projects that systematically map the literature available on a topic, identifying key concepts, theories, sources of evidence and gaps in the research” [16]. Scoping reviews are helpful when the literature in an area is complex and heterogeneous [16]. Since we do not know how many studies there are on this topic, or to what extent details of the training and support are reported, we consider a scoping review a suitable approach to adopt at this stage, particularly as synthesised research on the topic is limited. We conducted a preliminary search for existing scoping, and systematic reviews on this topic in the Joanna Briggs Institute’s (JBI) Database of Systematic Reviews and Implementation Reports, Cochrane Database of Systematic Reviews, CINAHL, and PubMed, but no relevant reviews were identified.

The objective of this scoping review is to comprehensively explore the literature and identify and map how facilitators have been trained for and supported in the facilitator role in implementation studies in health and community care. Specifically, the review aims to examine:

- What is reported in the literature regarding training and support of facilitators in terms of learning outcomes, content, dose, mode of delivery, pedagogical approaches, and qualifications of the trainers?
- How facilitators perceive the training and support they receive?

## Method

The scoping review will be conducted following the guidance of JBI [17] and reported in accordance with the Preferred Reporting Items for Systematic Reviews and Meta-Analyses extension for Scoping Reviews (the PRISMA-ScR) [18].

### Inclusion and exclusion criteria

Following the JBI guidance [17], the inclusion and exclusion criteria for the proposed review are categorised in terms of participants, concept, context, and sources (Table 1).

**Table 1:** Inclusion and exclusion criteria

	Inclusion criteria	Exclusion criteria
Participants	Individuals defined as facilitators support implementation processes Individuals with a similar role but named differently, e.g. coach or mentor, who explicitly use facilitation as a strategy for supporting implementation processes Facilitators support the implementation of innovations such as clinical guideline recommendations, treatments, and care programmes The target groups for facilitation are staff and managers in health and community care	Facilitation is only directed to education or training in specific clinical procedures Facilitators support the implementation of general quality improvement systems or similar initiatives
Concept	Training, education, supervision, and/or support provided to facilitators, or individuals in similar roles, is reported	
Context	The implementation takes place in health or community care	
Sources (types of studies)	Empirical studies or study protocols for empirical studies published in peer-reviewed journals in English, Swedish, Norwegian, and Danish	Review articles

### Search strategy

We have worked with an experienced information specialist at the Karolinska Institute Library and developed a search strategy that was informed by the strategy used by Cranley et al. [11]. Time span for searches in MEDLINE (Ovid), Embase (embase.com), Web of Science Core Collection, and CINAHL (Ebsco) is from inception to September 2020. The initial search strategy for MEDLINE (Ovid) is displayed in Table 2. In addition, we will review the reference lists of included articles. A secondary search to identify recently published articles will be performed prior to the completion of this review.

**Table 2:** Search strategy for MEDLINE (Ovid) and initial hits

1	(facilitator* or facilitative or facilitation).ti,ab,kf	57,253
2	facilitat*.ti	42,006
3	or/1–2	86,239
4	exp Evidence-Based Practice/	88,585
5	(ebp or ebm or ebn or cpg* or best practice*).ti,ab,kf	71,066
6	(evidence adj2 practice*).ti,ab,kf	17,982
7	(guideline* adj2 (implement* or adher*)).ti,ab,kf	9541
8	Guideline Adherence/	32,298
9	Quality Assurance, Health Care/	55,944
10	Benchmarking/	13,496
11	Guidelines as Topic/	40,056
12	Practice Guidelines as Topic/	119,138
13	(quality adj1 (improv* or manag*)).ti,ab,kf	74,492
14	exp Diffusion of Innovation/	19,994
15	((evidence or knowledge or research) adj2 ("use" or utili* or adopt* or implement* or disseminat* or uptake or transfer* or translat* or support)).ti,ab,kf	102,879
16	(innovation adj2 adopt*).ti,ab,kf	277
17	or/4–16	543,187
18	3 and 17	5499

### Study selection

Following the searches, duplicates will be excluded in EndNote using an automatic function. References will be transferred to Rayyan (https://rayyan.qcri.org/welcome) where the screening of titles and abstracts will be performed. The reviewers will ensure consistency in screening through conducting the following steps: (1) joint screening until all reviewers (*n* = 10) feel confident to start independent screening, (2) independent blinded screening of 25 titles/abstracts followed by a meeting and discussion to clarify inclusion/exclusion decisions, and (3) repetition of step 2 until an acceptable agreement is reached. For the subsequent process, the reviewers will be divided into five pairs. Each pair will screen a fifth of the titles/abstracts, independently and blinded from each other’s decisions. If consensus is not reached, the title/abstract will still be included not risking excluding an article that might fall within the inclusion criteria when the full text is reviewed.

To ensure consistency in reviewing full texts for inclusion/exclusion, a two-step process will be followed. In the first step, the full team will undertake joint screening of 20 full texts with discussion and clarification of inclusion criteria as appropriate. If needed, this will be repeated. Thereafter, four of the co-authors will assess full-text articles in two pairs, independently and blinded from each other’s decisions. For this process, we will use Covidence (https://www.covidence.org/). In the review of full-text articles, disagreements will be resolved by consensus in the pairs.

Throughout screening abstracts, full texts, and extracting data, the reviewers will have regular meetings to discuss and solve emerging issues.

### Data extraction

A preliminary data extraction form that is in line with the aims of this scoping review has been developed. Information extracted from the included full-text articles will comprise publication data, study details, details about facilitation and the facilitator(s), details on training and support and facilitator(s)’ perception of the training. The preliminary data extraction template is presented in Table 3. However, the extraction template may need to be refined as the review process progresses.

**Table 3:** Preliminary table for data extraction

Data extraction template	
Publication data	
	Authors(s):
	Year of publication:
Study details	
	Country
	Context (e.g. hospital, community care)
	Type of publication
	Innovation to be implemented
Details about facilitation and facilitator(s)	
	Overall definition of facilitation
	Cited source for facilitation approach
	Target group for facilitation
	Facilitator(s)’ position in the organisation
	Process of recruiting and appointing facilitator(s) and recruitment criteria
	Facilitator(s)’ background and qualifications
Details on training and support/supervision	
	Learning outcomes/goals of training
	Content of training (topics that are covered in the training)
	Dose (hours/days), number of sessions and time span (weeks/months) of training
	Mode of delivery of training (face-to-face, virtual, combined)
	Learning activities (e.g. lectures, interactive workshops, experiential training)
	Characteristics/qualifications of the trainer
	Learning outcomes/goals of support/supervision
	Content of support/supervision (topics that are covered in the support/supervision)
	Model for support/supervision (e.g. scheduled, on-demand)
	Dose (hours/days), number of sessions and time span (weeks/months) for support/supervision
	Mode of delivery of support/supervision (face-to-face, virtual, combined)
	Activities for supervision/support (e.g. lectures, reflection)
	Characteristics/qualifications of the provider of support/supervision
Facilitator(s)’ perception of the training and support/supervision	
	Method(s) used for evaluation of training
	Facilitator(s)’ perception of training
	Method(s) used for evaluation of support/supervision
	Facilitator(s)’ perception of support/supervision

Six of the co-authors, divided into three pairs, will conduct the data extraction. Each pair of co-authors will, independently and blinded from each other’s decisions, extract data from a third of the included full texts. The co-authors will start by extracting data in five articles. Thereafter, the extraction form will be evaluated based on knowledge from this initial work. If needed, the data extraction form will be revised. Modifications will be described in the complete scoping review paper. The tool Data Extraction 2.0 (https://www.covidence.org/) will be used for the data extraction process. Each pair of co-authors will meet and discuss differences in data extraction until they reach consensus. Throughout extraction of data, the reviewers will have regular meetings to discuss and solve emerging issues.

### Analysis

The extracted data on the details of training and support/supervision (see Table 3) and facilitator(s)’ perception of the training and support/supervision will be analysed to provide a comprehensive picture of how facilitator training and support is reported in the literature. The number and proportion of studies that report learning outcomes/goals of training (and/or support), content of training (and/or support), dose of training (and/or support), mode of delivery of training (and/or support), pedagogical approach of training (and/or support), characteristics/qualifications of the trainer (and/or supervisor), method(s) used for evaluation of training (and/or support), and facilitator(s)’ perception of training (and/or support) will be presented.

Qualitative data relating to learning outcomes/goals of training (and/or support), content of training (and/or support) pedagogical approach of training (and/or support), characteristics/qualifications of the trainer (and/or supervisor), mode of delivery of training (and/or support), and facilitator(s)’ perception of training (and/or support) will be analysed using an approach of inductive qualitative content analysis [19]. The extracted data for each item will be analysed in a process following three main phases: preparation, organising, and reporting. After reading through the data, open coding of the manifest content will be conducted, and the codes grouped into categories. Categories for each item will be created on a level that is meaningful in relation to the research question and the extracted data. Frequencies for the inductive categories will be reported. Furthermore, we plan to create categories of time intervals that reflect the extracted data on dose of training (and/or support). In addition, median, mean, minimum, and maximum dose (hours) will be calculated. Throughout the process of analysis, we will have a flexible approach and adapt the analyses to the extracted data.

### Presentation of the results

The results of the search strategy and selection process will be presented using a PRISMA flow diagram. The data on study details, facilitation and facilitator(s), and method(s) used for evaluation of training (and/or support) (see Table 3) will be presented in tables to provide contextual background for the findings on training and support of facilitators(s).

The categories created in the qualitative content analysis with frequencies will be presented in tables or figures. The tables and figures will be accompanied by text that provides details and rich descriptions of the content of the categories. We will also provide a narrative summary describing how the results relate to the review aim.

## Discussion

To our knowledge, this is the first scoping review of support and training for facilitators involved in the implementation of innovations in health and community care. This review aims to explore what has been reported in individual studies and then synthesise that knowledge.

Facilitation as an implementation strategy involves a facilitator who assesses and responds to characteristics of the innovation, the recipients, and the context. In turn, this places high demands on the facilitator’s abilities and skills [10]. To describe how facilitators have been trained for and supported in the role is therefore important. The proposed scoping review will contribute valuable knowledge on this topic by identifying and synthesising available literature. Furthermore, it will help to identify gaps in current research where further work is needed to promote appropriate training and support of implementation facilitators.

Selection of relevant literature may prove challenging in the conduct of the scoping review, not least because facilitation is a wide-ranging concept that encompasses both facilitator roles and facilitation processes. For example, facilitators are described in the literature relating to workshops or education sessions that are often stand-alone events rather than an ongoing process to support implementation. The training and support of such facilitators will most likely be different from the facilitator role that is the target for this scoping review. We will apply a careful process with joint screening to agree on inclusion and exclusion decisions. However, we will include rather than exclude papers if consensus is not reached, as exploration of the breadth of the literature is part of the scoping review approach [17].

We will not perform a quality assessment of included full texts. This is because firstly, scoping reviews generally aim to give an overview of the existing research on a specific topic regardless of the quality of the studies [17]. Secondly, we are not focusing on the outcomes of the studies, but rather on how a part of the research process is reported. Hence, a quality assessment is not considered relevant.

This scoping review will provide a systematic mapping of the literature regarding training and support of facilitators in implementation efforts and thereby contribute useful knowledge within implementation science to inform future facilitation initiatives.

## Data Availability

All data related to this protocol is available in this manuscript.

## Abbreviation

i-PARIHS: Integrated-Promoting Action on Research Implementation in Health Services
